# Maintaining a Surgery Service for Local Hospitals Under the Situation of a Decreasing Number of Surgeons in a Region of Japan

**DOI:** 10.1007/s00268-014-2779-5

**Published:** 2014-09-13

**Authors:** Joji Watanabe, Hiroaki Saito, Shinji Otani, Masahide Ikeguchi

**Affiliations:** Division of Surgical Oncology, Department of Surgery, Tottori University Hospital, 36-1 Nishi-cho, Yonago, Tottori 683-8504 Japan

## Abstract

**Background:**

The number of surgeons is decreasing in Japan, leading to the problem of how to maintain a surgery service in local hospitals. We introduce our strategy for supporting ongoing surgical services in regional hospitals by dispatching surgeons temporarily to assist in operations.

**Methods:**

We conducted a questionnaire-based survey at three local hospitals in Tottori and a neighboring prefecture to which surgeons from our department were temporarily dispatched over 5 years from January 2008 to March 2013.

**Results:**

We supported 686 operations at three hospitals over 5 years. The average age of the patients was 72.4 years. Of the diseases treated, 45.1 % were malignant, and 54.9 % were benign. The emergency operation rate was 17.3 %.

**Conclusions:**

Our strategy has produced a continuous surgical service at local hospitals in the face of diminishing numbers of surgeons. We recommend that such a strategy be adopted in other regions in which there are a decreasing number of surgeons and where it is not easy to move patients elsewhere for care.

## Introduction

In Japan, the number of doctors amounts to two-thirds of the average number of clinicians in countries belonging to the Organization for Economic Cooperation and Development. Although the Japanese government has encouraged an increase in the number of doctors, there remains an inappropriate number within every medical department. The number of surgeons in Japan is thus also decreasing. Surgeon dispatch systems are controlled by the departments of surgery of medical universities.

Many medical university departments of surgery tend to attract surgeons away from local hospitals to practice in more densely populated urban areas. Each operation requires at least two surgeons and sometimes more than three for laparoscopic surgery. Maintenance of a continuous, stable surgery service in district hospitals is a growing problem.

**Fig. 1:**
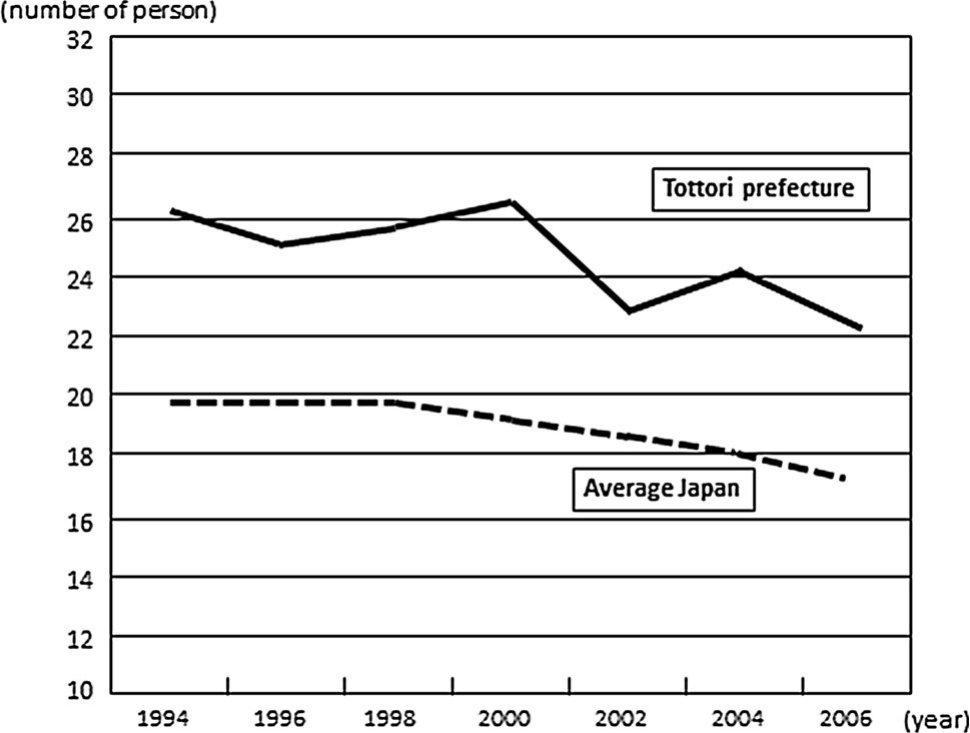
Number of surgeons per 100,000 people in Japan. Change in number of surgeons in Tottori Prefecture (*solid line*) and all-Japan average (*dashed line*). Adapted from Ref. [1]

**Fig. 2:**
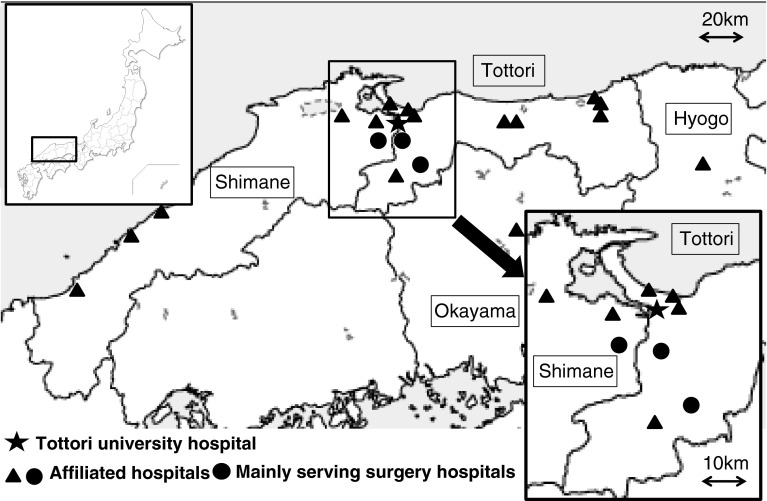
Location of Tottori University Hospital and local hospitals. Location of hospitals with surgery service is limited to those near the university hospital

Our hospital is located in Tottori Prefecture, one of the most sparsely populated areas in Japan. In Tottori, the number of elderly persons is increasing while the population as a whole is decreasing. The number of surgeons per 100,000 population in Tottori prefecture remains higher than the average for Japan (Fig. 1). At present, however, it has reached its lowest level [1], thereby leading to a detrimental effect on surgical services at local hospitals. Our department in Tottori University Hospital delivers gastrointestinal surgery and pediatric surgery services to Tottori and affiliated neighborhood hospitals nearby (Fig. 2). We describe our strategy for supplementing the surgery service in surrounding local hospitals by dispatching surgeons to assist in operations.

## Methods

We have devised a system that delivers assistance at any time and to any affiliated local hospital by following the necessary regulatory procedures established at Tottori University. A local hospital contacts our department to request that a surgeon or surgeons be dispatched when temporary support is needed for performing surgical procedures. We conducted a questionnaire-based survey in the three surrounding local hospitals that are the main recipients of temporary surgeons from our department. It covered a period of 5 years, from January 2008 to March 2013. These placements included periodic support for operations in one hospital and temporary support in the other two. Our data included the number of surgeons, number of operations, number of supported operations, number of emergency operation, age of the patients, and disease characteristics. We calculated the average number of full-time employee surgeons and average number of director or assistant directors (0.5). The hospitals could be reached by mountain roads within approximately 1 h.

## Results

We supported 686 operations at four hospitals over 5 years. The male/female ratio was 1:1, and the average age was 72.4 years. Of the diseases treated, 45.1 % were malignant and 54.9 % were benign. A total emergency operation rate for temporary support was 23.9 %.

Hospital A has 199 beds, an average of 2.5 surgeons, and an average of 164.8 operations per year. The rate of our support in all operations was 37.5 %, the rate of emergency surgery in our support operations was 13.8 %. Hospital B has 198 beds, an average of 1.6 surgeons, and an average of 60.4 operations per year. The rate of our support in all operations was 65.2 %. The rate of emergency surgery in our support operations was 25.4 %. Hospital C, which enjoyed periodic support for operations, has 99 beds, an average number of 1.1 surgeons, and an average of 81.4 operations per year. The rate of our support in all operations was 59.5 %. The rate of emergency surgery in our support operations was 66 % (Table 1).

**Table 1:** Surgical services at local hospitals

Hospital	No. of beds	Average no. of surgeons	Average no. of operations (per year)	No. of supported operations (% supported)	No. of emergency operations (rate for temporary supports)	Average age of patients (years)	Character of disease	%
A	199	2.5	164.8	247 (37.5 %)	34 (13.8 %)	70.9	Malignant Benign	59.1 40.9
B	198	1.6	60.4	197 (65.2 %)	50 (25.4 %)	70.4	Malignant Benign	31.5 68.5
C	99	1.1	81.4	242 (59.5 %)	35 (66.0 %)	74.2	Malignant Benign	26.4 73.6
Total				686	119 (23.9 %)	72.4	Malignant Benign	45.1 54.9

Director or assistant director of hospital was defined as 0.5 person

Hospital A was investigated for 4 years from April 2009 to March 2013. Hospital B was investigated for 5 years from April 2008 to March 2013. Hospital C was investigated for 5 years from January 2008 to March 2013. These data include periodic support operations

## Discussion

**Fig. 3:**
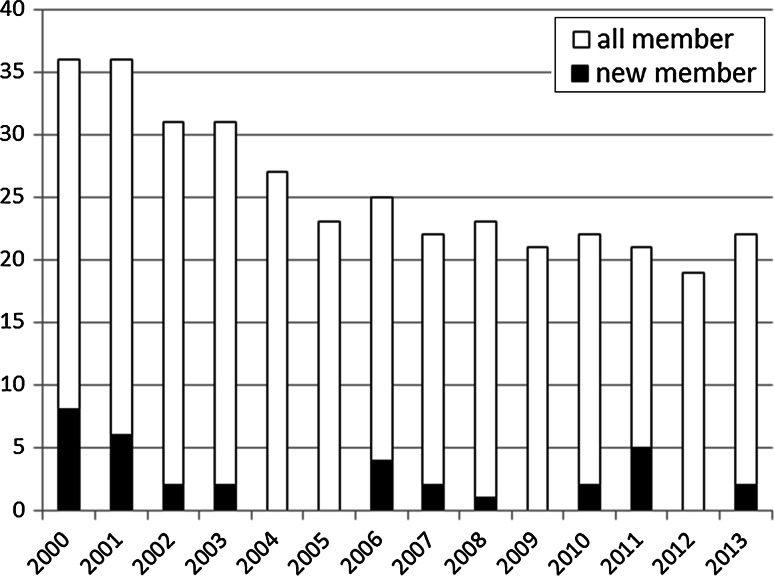
Changes in the number of staff at the 1st Department of Surgery, Tottori University. The decreasing surgeons in Japan influence the number of our staff members. There were more than 35 office members in 2000. By 2012, this number had decreased to fewer than 20

In 2008, the total number of doctors in Japan was 1.19-fold that of 1994. In contrast, the number of surgeons decreased by 0.87-fold during the same period [2]. This shows that the number of young doctors who wish to become surgeons is decreasing. Our office staff in 2000 numbered more than 35 people but has been declining yearly, with fewer than 20 in 2012 (Fig. 3). The average age of surgeons in local hospitals is rising, and more are facing retirement age. In recent years, fewer young surgeons have signed up, and the total number of surgeons in our department continues to decrease. Therefore, it is becoming more difficult to dispatch surgeons to local hospitals. Typically, there is limited time in which to prepare for an operation at a local hospital, so it is advantageous for both patients and surgeons to make fullest use of existing resources within that hospital. We consider it of great importance to maintain a hospital’s current resources because once a surgical unit is closed it is very difficult to resume activity.

The requirements of the hospitals to which our surgeons were dispatched varied. Hospital A, which is the major one in the area, performs many procedures for malignant disease. On the other hand, hospitals B and C attend more closely to the needs of the general populace in performing emergent surgery and procedures for acute and benign disease. If these operations were not possible in these locations, such patients would have to be transferred to city hospitals. The surgical capacity in city areas is full at present. Also, many patients in local hospitals are elderly, and they wish to be treated in their home area. Surgeons participating in our plan can make extra income for helping. The local hospitals who participate gain income for their hospitals. Thus, this surgeon delivery system benefits patients, surgeons, and hospitals. Most of the surgeons who have requested assistance and those who gave it at different hospitals understood the local hospital situation and believed this system to be suitable for the time being. An additional asset of our strategy is that as well as providing support for operations, our surgeons deliver coaching sessions in surgical techniques to the local hospitals’ surgeons, thus improving overall standards in high-quality surgery.

Because the doctor-dispatch system depends on medical departments in Japan, it is difficult to solve the problem of how to maintain surgical services in local hospitals for short periods. Various methods of supporting local hospital surgery in Japan have been reported. Nakano et al. [3] reported the system of telephotograph transfer to help with decision-making regarding indications for surgery. However, this system is expensive and cannot be performed locally. Morimoto and Shimada [4] reported clinical pathway coordination between high-volume centers and local hospitals. This system does not include all levels of cancer, being targeted to less than stage II. This system is limited by there being insufficient surgeons for specific operations. Yoshida and Kumakura [5] suggested a medical symposium and discussion for medical students who might pledge to return to their home town from their teaching hospital after qualifying. However, this involves a long waiting period before the student becomes a fully qualified doctor. Although these are all valid suggestions, a method is needed to deliver adequate surgery in the face of the limited number of present-day surgeons.

Some organizations provide surgical volunteers worldwide [6–9], whereby surgeons from technologically advanced areas volunteer to use their expertise in underserved regions. Meier [10] reported that volunteer surgeons can make significant contributions in poorly resourced areas. The situation is similar to that in Japan, where the number of surgeons is decreasing in an area where it is not easy to transfer patients to another hospital. However, these systems are targeted to developing countries. In Japan, we require a surgeon-dispatching system for the future along the lines of the strategy discussed herein.

Other problems include the fact that movement by surgeons tends to be limited to within a specific area at or near a university hospital, whereas many local hospitals are located in more remote areas. Surgeons also have limited time and access to transport. It will be necessary to make maximal use of our existing doctor-dispatch system and devise another system to deliver surgery to locations much farther away from the university hospital area. Furthermore, the situation differs in various regions. A standard and suitable surgery outreach system is needed for all surgeon-decreasing areas in which it is not easy to move transfer patients from one hospital to another.
